# Metabolite Profiling of *Helichrysum italicum* Derived Food Supplements by ^1^H-NMR-Based Metabolomics

**DOI:** 10.3390/molecules26216619

**Published:** 2021-10-31

**Authors:** Antonietta Cerulli, Milena Masullo, Sonia Piacente

**Affiliations:** Dipartimento di Farmacia, Università degli Studi di Salerno, Via Giovanni Paolo II n. 132, 84084 Fisciano, SA, Italy; acerulli@unisa.it (A.C.); mmasullo@unisa.it (M.M.)

**Keywords:** green extractions, *Helichrysum italicum* derived food supplements, NMR metabolomics, multivariate data analysis

## Abstract

*Helichrysum italicum* is a medicinal plant from the Mediterranean area, widely used in traditional medicine for its anti-inflammatory, antibacterial and antioxidant properties and for its preventive effects on microcirculation diseases. Due to these properties, it finds large applications in cosmetic, food and pharmaceutical fields. Additionally, hydroalcoholic extracts and mother tinctures based on *H. italicum* represent products with a high commercial value, widely distributed not only in drug stores but also on on-line markets. The different extraction procedures used can greatly affect the fingerprints of the extracts, resulting in a different qualitative or quantitative profile of the chemical constituents responsible for biological activity. The aim of the present study was to characterize the composition of bioactive compounds present in water-ethanol and glycerol extracts of *H. italicum* derived food supplements. Metabolite profiles of the extracts were obtained by ^1^H NMR experiments and data were processed by multivariate statistical analysis to highlight differences in the extracts and to evidence the extracts with the highest concentrations of bioactive metabolites. In detail, this work highlights how derived food supplements of *H. italicum* obtained using ethanol-water mixtures ranging from 45% to 20% of ethanol represent the products with the highest amount of both primary (amino acids) and secondary metabolites including 3,4-dicaffeoylquinic acid (**9**), chlorogenic acid (**10**), 3,5-dicaffeoylquinic acid (**11**), and kaempferol 3-*O*-glucopyranoside (**12**). Moreover, it is evident that the use of an ethanol-water mixture 20:80 is the most suitable method to afford the highest number of phenolic compounds, while food-derived supplements obtained by glycerol extraction are characterized by a high amount of β-glucose and α-glucose and a low content of phenolic compounds.

## 1. Introduction

Plant materials are extremely complex matrices due to the presence of chemical constituents belonging to different classes [1]. Differences in the production and accumulation of primary and secondary metabolites can be due to seasonal differences related to phenology or environmental changes in the biotic and abiotic factors, to geographical differences involving various populations (genetic differences within a plant species), or to environmental conditions of the growth location of the individual species, especially when they have genetic homogeneity (i.e., cultivars) [2].

Enriched fractions or pure phytochemicals must be obtained via extraction [1]. Extraction procedures often involve different steps and the use of unsustainable solvents which can interfere with the presence of chemical constituents [3]. In recent years, new technologies and methods of extraction occurred which accelerated the extraction and analysis of phytochemicals [3,4], along with an increasing interest in the development of green and environmentally friendly extraction methods [5,6,7]. Food supplements are generally extracted with atoxic solvents, such as ethanol and water.

Ethanol is the most common bio-solvent, completely biodegradable, obtained by the fermentation of sugar-rich materials such as sugar beet and cereals [5]. Therefore, green solvents such as water and aqueous ethanol solutions are among the preferred ones for extraction processes [8]. Aside from these, glycerol also represents an interesting solvent for extracting constituents from plants without the use of alcohol [1].

*Helichrysum italicum* is a medicinal plant from the Mediterranean area, widely used for its anti-inflammatory, antibacterial and antioxidant properties and for its preventive effects on microcirculation disease. Due to its dermofunctional, antiallergic and antieczematic activities, *H. italicum* extracts are also employed in cosmetic and pharmaceutical sectors. *H. italicum* as a decoction is used to soothe cough, to help bronchial mucus expectoration, and to soothe allergies caused by the inflammation of mucous membranes of the nose [9,10]. Additionally, hydroalcoholic extracts and mother tinctures based on *H. italicum* represent products with a high commercial value, widely distributed not only in drug stores but also on on-line markets.

Several analytical studies were carried out on *H. italicum*, mainly focused on its essential oils, but few reports investigated the metabolite composition of the polar extracts represented by commercial products such as hydroalcoholic extracts and mother tinctures [9,11,12,13].

The aim of the present study was to compare the chemical composition of water-ethanol extracts and glycerol extracts of *H. italicum* derived food supplements by ^1^H NMR analysis, since the extraction procedures can greatly affect the fingerprints of the extracts, resulting in a different qualitative or quantitative profile of the chemical constituents responsible for biological activity [14,15]. ^1^H NMR is a powerful analytical tool in the field of quality evaluation of various food and medicinal plants since it is a non-destructive technique and is characterized by a simple sample preparation and rapid analysis [14,16]. It can simultaneously identify diverse groups of secondary metabolites as well as abundant primary compounds. Furthermore, ^1^H NMR can provide comprehensive characteristic fingerprints of herbal products and is widely used to analyze the plant metabolite composition influenced by extraction methods. In this context, metabolomic approaches based on NMR spectroscopy may help to identify and estimate the relative abundance of metabolites. Metabolomic analysis generates huge datasets that make necessary the application of chemometric methods [14,17,18]. Thus, in this work, an approach based on NMR metabolomics with multivariate data analysis (MVDA) was used to identify the metabolite variation among hydroalcoholic and glycerol extracts based on *H. italicum*.

## 2. Results and Discussion

### 2.1. Untargeted Metabolite Profiling of Helichrysum italicum Derived Food Supplements

Herbal preparations constitute very complex matrixes that encompass a great number of metabolites such as primary metabolites, including amino acids, carbohydrates and lipids as well as secondary metabolites, also known as specialized metabolites, comprising phenolic compounds, terpenes and alkaloids.

**Figure 1:**
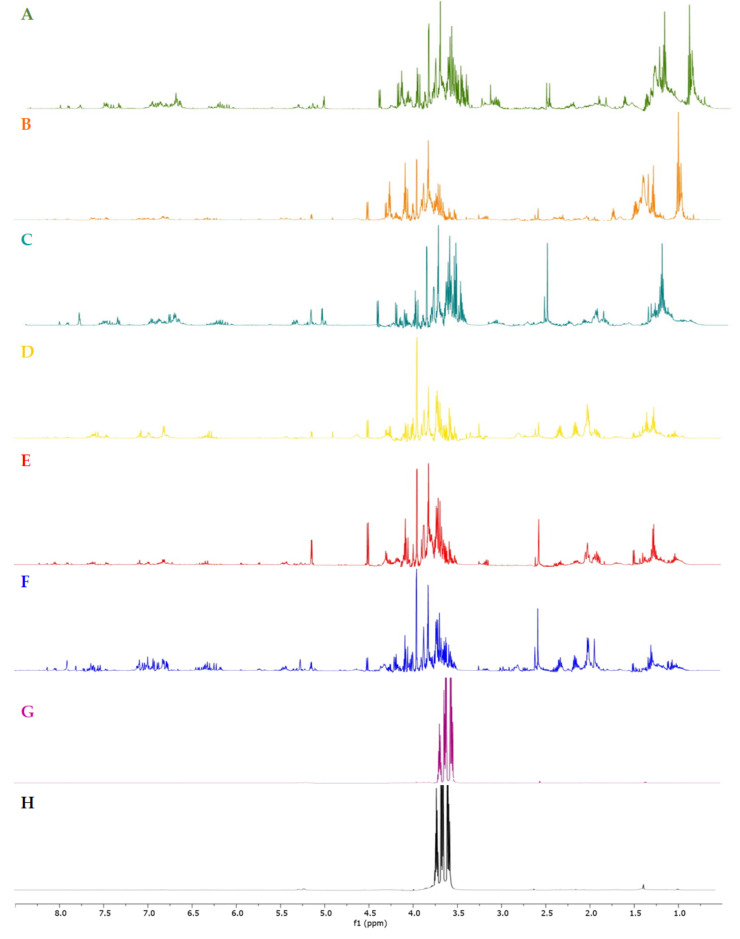
^1^H NMR spectra of the derived food supplements (**A**–**H**) of *H. italicum* (**A**: 60:40; **B**: 50:50; **C**: 46:54; **D** and **E**: 45:55; **F**: 20:80 EtOH-H_2_O extracts; **G**: glycerol extract; **H**: water-glycerol extract).

The ^1^H NMR profiles of derived food supplements based on *H. italicum* flowers were compared (Figures S1–S3, S7, S11, S12, S17 and S18, Supplementary Materials). These included commercial preparations obtained with EtOH-H_2_O at different concentrations (**A**: 60:40, **B**: 50:50, **C**: 46:54, **D** and **E**: 45:55, and **F**: 20:80), solvents commonly known to have a good capacity for the extraction of secondary metabolites; a preparation obtained by glycerol extraction (**G**) and a further one obtained using water with the addition of glycerol (**H**). The ^1^H NMR spectra were observed to be crowded, with several overlapping signals, which could be divided in three regions: aliphatic, carbinol and aromatic protons (Figure 1). Firstly, principal component analysis (PCA) was used in order to gain an overview on trends and outliers among the samples. Unsupervised PCA data analysis was performed starting from NMR peak lists obtained from the entire spectrum range (8.5–0.5 ppm) of samples and by measuring the selected peak area in the ^1^H NMR spectra (Figure 1). Signals corresponding to water, methanol and glycerol residues were excluded. A matrix was obtained by using these areas (variables), while the columns of the matrix were the different commercial food supplements (observations).

The first component explained the 38.9% of variance while the second one the 20.1%. The choice of principal components was established on the basis of the fitting (R2X) and predictive (Q2X) values for the PCA.

**Figure 2:**
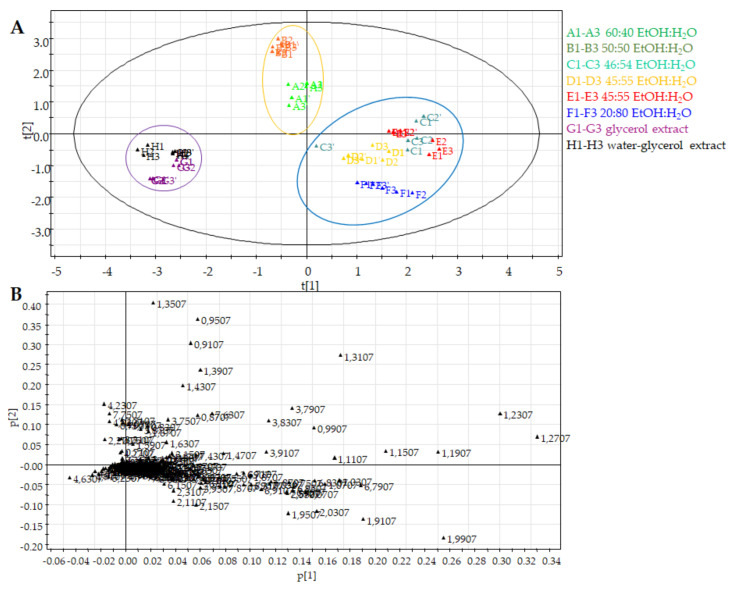
Principal component analysis of *H. italicum* derived food supplements (**A**: 60:40; **B**: 50:50; **C**: 46:54; **D** and **E**: 45:55; **F**: 20:80 EtOH-H_2_O extracts; **G**: glycerol extract; **H**: water-glycerol extract) obtained by untargeted analysis. (**A**) PCA score scatter plot; (**B**) PCA loading plot.

The untargeted PCA score plot (Figure 2A) showed three different clusters. The first cluster is for tinctures **A** and **B** that were obtained with the highest percentage of EtOH; the second cluster was characterized by commercial preparations obtained using a percentage of EtOH in the range of 46% to 20% (**C**, **D**, **E** and **F**) and the last cluster contained food supplements prepared without ethanol solvent (**G** and **H**). Therefore, the PCA score scatter plot clearly discriminated the different extraction methods used for fresh flowers of *Helichrysum italicum*.

The PCA loading plot highlighted the signals responsible for the distribution on the PCA score plot. Figure 2B showed that clustering of the lower polarity extracts (**A** and **B**), characterized by a main presence of EtOH respective to the other derived food supplements, was due to the signals at δ 0.91, 0.95, 1.31 and 1.35 ascribable to a main presence of fatty acids. Moreover, the PCA loading plot of the hydroalcoholic extracts **C**–**F** showed signals falling in the aromatic region, corresponding to phenolic compounds. These data were in agreement with the capacity of EtOH-H_2_O mixtures to extract secondary metabolites responsible of *Helichrysum italicum* biological properties.

Thus, water can play an important role in the swelling of plant material, whereas ethanol is responsible for disrupting the binding between the solutes and plant matrix, thus enabling better mass transfer of the compounds. The mixtures of EtOH-H_2_O, characterized by a main percentage of water, showed the better synergistic effect for phenolic extraction.

The ^1^H NMR spectra of samples **G** and **H** showed intense signals due to glycerol (Figure 1), which were excluded in the PCA analysis. Figure 2B showed that this last clustering was characterized by a main presence of signals falling in the sugar region.

### 2.2. Metabolite Fingerprinting by 1D and 2D NMR Spectroscopic Analysis

**Figure 3:**
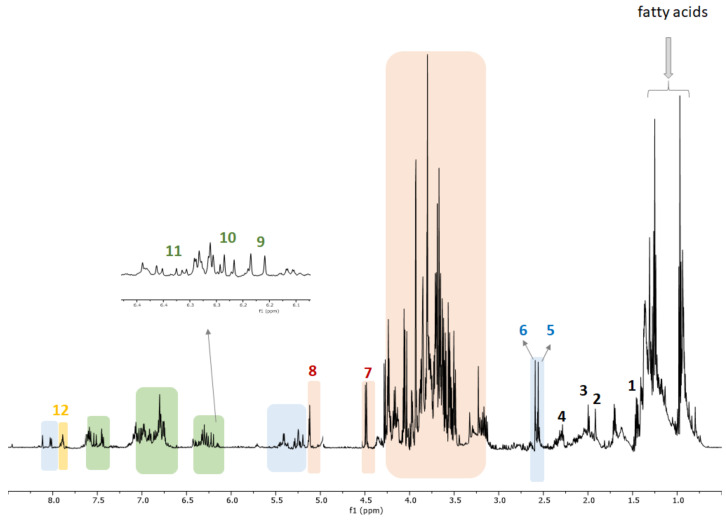
^1^H NMR spectrum with annotations of identified metabolites detected in *H. italicum*: Alanine (**1**); GABA (**2**); lysine (**3**); valine (**4**); 12-hydroxytremetone (**5**); gnaphaliol (**6**); β-glucose (**7**); α-glucose (**8**); 3,4-dicaffeoylquinic acid (**9**); chlorogenic acid (**10**); 3,5-dicaffeoylquinic acid (**11**); kaempferol 3-*O*-glucopyranoside (**12**).

**Figure 4:**
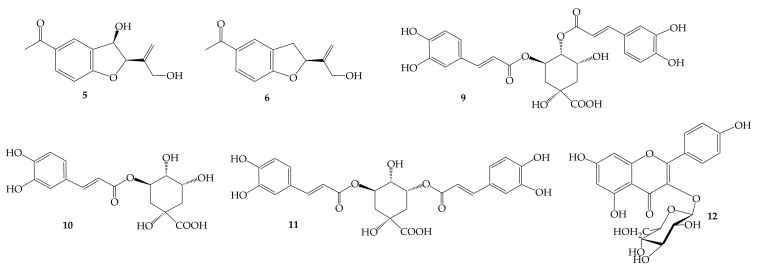
Secondary metabolites identified in derived food supplements of *H. italicum*.

^1^H NMR metabolomics has the advantage of observing signals of different types of primary and secondary metabolites simultaneously. The biological activity of *H. italicum* derived food supplements is attributed to the presence of natural products such as phenolics and flavonoids derivatives [9,10]. Previous investigations on *H. italicum* flowers led to the isolation of caffeoyl quinic derivatives, flavonoids and acetophenone compounds [9,10]. To discriminate how the different extractions can affect the content of metabolites responsible for the biological activity, a targeted principal component analysis was carried out. Therefore, an accurate analysis of the proton spectra was performed to assign unambiguously a key signal characteristic of each metabolite, thus a dataset of ^1^H NMR characteristic signals of each metabolite has been produced. In this way, 12 metabolites have been assigned; in detail 6 primary metabolites, namely: alanine (**1**), GABA (**2**), lysine (**3**), valine (**4**), β-glucose (**7**), α-glucose (**8**), and 6 secondary compounds namely 12-hydroxytremetone (**5**), gnaphaliol (**6**), 3,4-dicaffeoylquinic acid (**9**), chlorogenic acid (**10**), 3,5-dicaffeoylquinic acid (**11**), and kaempferol 3-*O*-glucopyranoside (**12**) were identified (Figure 3 and Figure 4).

The unambiguous identification of the metabolites in the ^1^H NMR spectra of derived food supplements was further confirmed by the analysis of the 2D NMR spectra. 

^1^H NMR analysis of derived food supplements allowed to identify primary metabolites such as aminoacids and carbohydrates. Typical signals at δ 1.48 (d, *J* = 7.2 Hz), 1.92 (t, *J* = 7.5 Hz), 2.00 (m) and 2.31 (m) ascribable to alanine (**1**), γ-aminobutyric acid (GABA) (**2**), lysine (**3**) and valine (**4**) were evident. The ^1^H NMR spectra displayed signals of two anomeric protons related to β-glucose (4.50, d, *J* = 8.0 Hz) (**7**) and α-glucose (5.14, d, *J* = 3.6 Hz) (**8**) (Table 1).

Moreover, ^1^H NMR spectra highlighted the presence of three aromatic protons of a typical ABX system, two protons linked to oxygenated carbons, one alcoholic primary function, two geminal olefinic protons and acetyl groups, suggesting the presence of groups ascribable to the acetophenone skeleton occurring in characteristic compounds previously isolated from *H. italicum*. In particular, acetyl groups at δ 2.56 (s) and δ 2.60 (s) were attributed to acetophenone 12-hydroxytremetone (**5**) and gnaphaliol (**6**), respectively.

The aromatic region of NMR spectra showed several signals attributed to caffeoyl quinic acids and flavonoid derivatives by comparison with spectra of each pure compound isolated in our previous investigations [9,10]. In particular, ^1^H NMR data displayed different sets of doublets with a coupling constant of 16.0 Hz, typical of trans-olefinic groups [19,20], corresponding to caffeoylquinic derivatives [21,22]. In detail, the signals at δ 6.23, 6.31, and 6.37, were chosen as key signals for 3,4-dicaffeoylquinic acid (**9**), chlorogenic acid (**10**) and 3,5-dicaffeoylquinic acid (**11**), respectively (Table 1) [23]. Moreover, the signal at δ 7.87 (2H, d, *J* = 8.2 Hz) was attributed to H-2′ and H-6′ and was chosen as the characteristic signal to identify kaempferol 3-*O*-glucopyranoside (Table 1) [24].

### 2.3. Targeted Multivariate Statistical Analysis

In this study, ^1^H NMR derived datasets were subjected to PCA to understand the clustering characteristic of the commercial preparations. The resulting model, obtained after scaling data by Pareto scaling, showed good fitness and the absence of outliers. The result of the validation test further emphasized the significance and predictability of the model when the targeted approach was applied; in particular, PC1 contributed to 52.5% of the variance, followed by PC2, which contributed to 21.4%. The first two PCs exhibited a total variance of 73.9%. Therefore, the extracts were well discriminated from each other.

**Figure 5:**
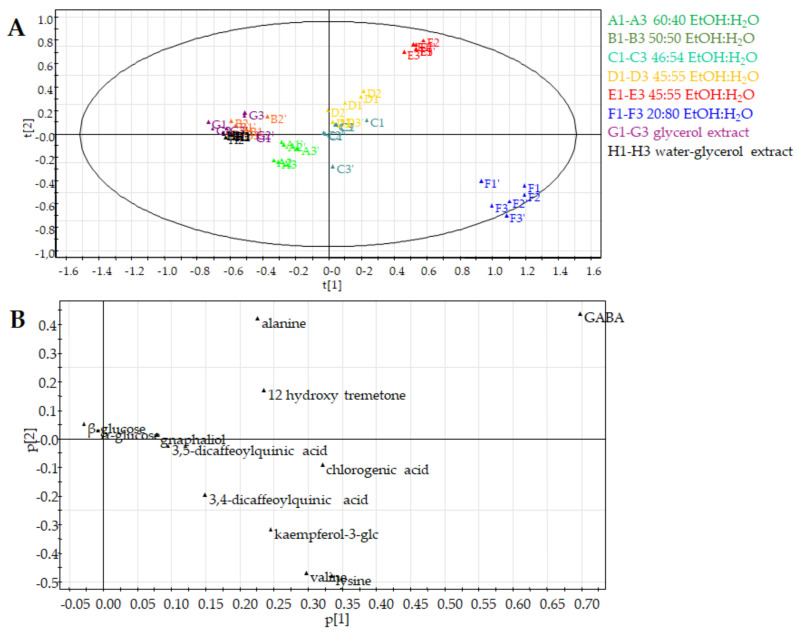
Principal component analysis of *H. italicum* derived food supplements (**A**: 60:40; **B**: 50:50; **C**: 46:54; **D** and **E**: 45:55; **F**: 20:80 EtOH-H_2_O extracts; **G**: glycerol extract; **H**: water-glycerol extract) obtained by targeted analysis. (**A**) PCA score scatter plot; (**B**) PCA loading plot.

The PCA score plot showed the separation of commercial products into clusters (observations) (Figure 5A). The PCA loading plot allowed to highlight metabolites responsible for the discrimination between different food supplements (Figure 5B and Figure S19, Supplementary). In detail, the PCA loading plot highlighted that all metabolites except β-glucose (**7**) and α-glucose (**8**) discriminated observations through the first principal component (Figure 5B). Secondary metabolites such as dicaffeoylquinic acid derivatives (**9** and **11**), chlorogenic acid (**10**), and kaempferol-3-*O*-glucopyranoside (**12**) as well as amino acids such as lysine and valine were responsible for the distribution of extracts along the second principal component. On the basis of the PCA targeted analysis, and considering the number of phenolic compounds, samples **C**–**F** showed a higher content of compounds **10**–**12** than the other food supplements, and among them, sample **F** showed the highest amount (Figure S19, Supplementary Information).

As reported in literature, the biological activities shown by *H. italicum* flower extracts are due to phenolic compounds [10,11]. Among these, caffeoylquinic acid derivatives (**9**–**11**) can be considered the main compounds occurring in the derived food supplements. Caffeoylquinic acids represent an important group of phenolic acids with potential health benefits, made up of quinic acid and one to four residues of caffeoyl groups [25,26]. They are widely found in a variety of plants as well as in different foods, such as vegetables, fruits, spices and coffee. Literature data provide strong evidence on their wide range of bioactivities, including antioxidant, antibacterial, antiparasitic, neuroprotective, anti-inflammatory, anticancer, antiviral and antidiabetic [25]. Chlorogenic acid (**10**) is one of the main polyphenols in the human diet, and it possesses many health-promoting properties. It has been approved by the China Food and Drug Administration (CFDA) as an anticancer drug, and most research regards its health benefits on disorders related to metabolic syndrome [27].

Results obtained by PCA targeted analysis are in agreement with PCA untargeted analysis; in fact, in both cases a higher abundance of secondary metabolites in hydroalcoholic food supplements has been observed if compared to products obtained by glycerol extraction.

**Figure 6:**
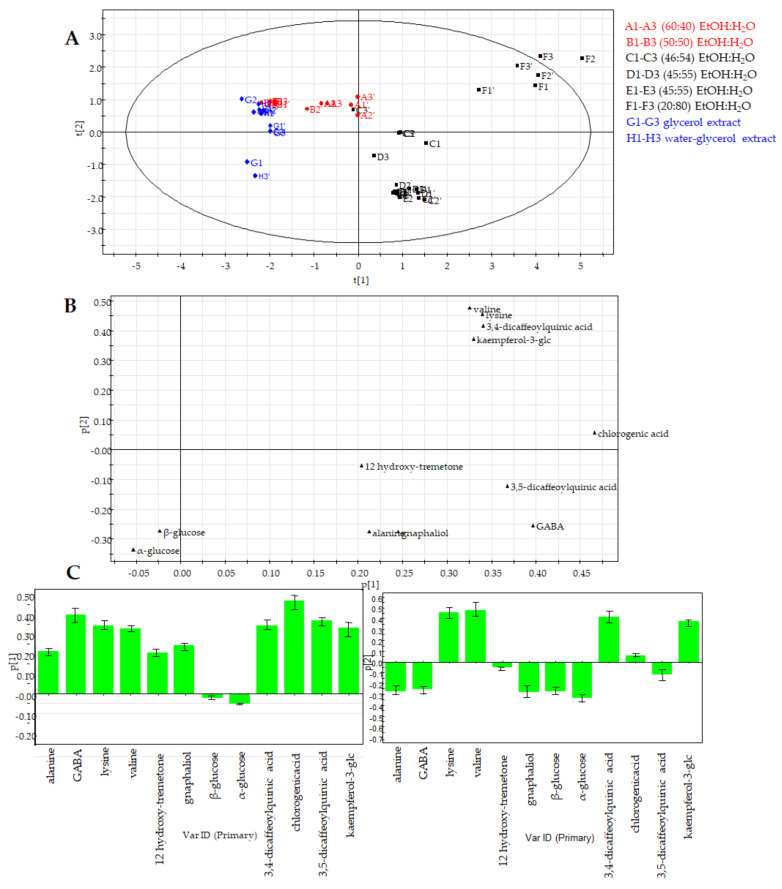
PLS-DA of *H. italicum* derived food supplements (**A**: 60:40; **B**: 50:50; **C**: 46:54; **D** and **E**: 45:55; **F**: 20:80 EtOH-H_2_O extracts; **G**: glycerol extract; **H**: water-glycerol extract) obtained by targeted analysis. (**A**) PLS-DA score scatter plot; (**B**) PLS-DA loading plot; (**C**) PLS-DA loading column plot.

Moreover, a discriminant classification was carried out using PLS-DA, a method based on the PLS regression algorithm that uses arbitrary classes as Y for the regression. Figure 6 shows the comparative PLS-DA analysis of extracts classified by ethanol-water mixtures with a higher percentage of ethanol (**A** and **B**), lower percentage of ethanol (**C**–**F**) and without ethanol (**G** and **H**). The PLS-DA score plot with component one explained 38% of the variation and with component two 17%, exhibiting a good separation between the groups. The PLS-DA analysis showed a distinct separation (R^2^Y, 0.51) and a good predictability (Q^2^, 0.49). The model was validated by cross-validation techniques and permutation tests according to standardized good practice to minimize false discoveries and to obtain robust statistical models.

PLS-DA showed a main separation among extracts **C**, **D**, **E** and **F**, characterized by ethanol-water mixtures ranging from 46:54 to 20:80, and the other extracts along the first principal component (PC1) (Figure 6A). In Figure 6B, the loading scatter plot shows significant metabolites based on contributions and reliability to the separation observed in the score scatter plot. Metabolites in the loading plot that are distant from the origin can be considered as markers of the derived food supplements as a confirmation of their different distribution in different samples (Figure 6B). In addition, the specific contribution of single variables to the principal components are reported in Figure 6C. In the column loading plot along PC1, metabolites marked above the baseline are present in the highest concentrations in the extracts **C**-**F**. Further information obtained by the contribution of compounds to PC2 highlighted how the extraction with 20:80 EtOH-H_2_O for the derived food supplement F seems to determine higher concentrations of primary metabolites as amino acids (valine and lysine), and of some secondary metabolites 3,4-dicaffeoylquinic acid (**9**), chlorogenic acid (**10**) and kaempferol 3-*O*-glucopyranoside (**12**) if compared to the other solvents.

## 3. Materials and Methods

### 3.1. Plant Material and Sample Preparation

Two batches of eight commercial food supplements, obtained from fresh flowers, were purchased from different herbal companies. In detail, commercial preparations obtained with EtOH-H_2_O at different concentrations **A**: 60:40, **B**: 50:50, **C**: 46:54, **D** and **E**: 45:55, **F**: 20:80, a preparation obtained by glycerol extraction (**G**), and a further one using water with the addition of glycerol (**H**), were analyzed. In this case, 3 mg of each derived food supplement, prepared in triplicate, were dissolved in 550 µL MeOH-d_4_ (purchased from Sigma-Aldrich, Darmstadt, Germany) transferred in 5 mm NMR tubes. Successively all the samples were used for ^1^H NMR analysis.

### 3.2. Generation of ^1^H NMR Metabolic Profiles

NMR experiments were acquired on a Bruker DRX-600 spectrometer (Bruker BioSpin GmBH, Rheinstetten, Germany) equipped with a Bruker 5 mm TCI CryoProbe at 300 K. All NMR spectra were acquired in methanol-*d*_4_ (99.95%, Sigma-Aldrich) and standard pulse sequences and phase cycling were used for DQF-COSY, HSQC, and HMBC spectra. The NMR data were processed using TopSpin 3.2 software. The analysis temperature was 24 °C. The relaxation delay was 4.0 s, and the acquisition time was 3.3 s. Spectra were the result of 256 scans, with data collected into 64 k data points. Each free induction decay (FID) was zero-filled to 128 k data points. Prior to Fourier transformation, an exponential window function with a line broadening factor of 0.2 Hz was applied. HSQC was obtained with a spectral width of 10 ppm and 150 ppm in the proton and carbon dimensions, respectively, 1 K data points, 64 scans, 256 t1 increments and a recycle delay of 2 s. HMBC was obtained with a spectral width of 10 ppm and 220 ppm in the proton and carbon dimensions, respectively, 4 K data points, 120 scans, 256 t1 increments and a recycle delay of 2 s.

### 3.3. NMR Data Processing

The spectra were imported into the MestreNova 10 software. All spectra were manually phased and baseline corrected. Spectra were referenced using the solvent signal at 3.34 as the chemical shift standard, obtaining good peak alignment. Bucketing was performed within 0.5–8.5 ppm region (spectral buckets of 0.004 ppm), excluding the signals of the residual non-deuterated methanol, deuterated methanol and water. The obtained data set was normalized by total sum normalization. Finally, the spectra were converted to ASCII format. Each sample was analyzed in triplicate. Identification of metabolites was achieved using chemical shifts known for each compound. In this way, each bucket corresponds to a defined signal or to a group of signals, which simplifies the interpretation of the statistical results.

### 3.4. Multivariate Data Analysis (Principal Component Analysis)

For the untargeted approach, chemical shifts deriving from ^1^H NMR were evaluated using ASCII derived by analysis of samples on MestreNOVA 10. After exporting the processed data in tabular format (.csv file), further analyses of the data matrix were performed by SIMCA-P þ software 12.0 (Umetrics AB, Umea, Sweden) by principal component analysis (PCA). The PCA was performed by applying chemical shifts obtained from NMR analysis. In this first step, PCA was employed in order to acquire a general insight and visualize any relation (trends, outliers) among the observations (samples).

For the targeted approach, a matrix was obtained starting by selected chemical shifts corresponding to specific primary and secondary metabolites, constituted by 12 variables and 8 observations. The resulting metabolomics data were processed using SIMCA P_+_ software 12.0 (Umetrics AB, Umea, Sweden) by PCA in order to identify similarities among our samples. A PLS-DA-based method was applied to discriminate the samples based on the extraction solvent. In the protocol here followed, discriminant classification was carried out using PLS-DA, a method based on the partial least squares (PLS) regression algorithm giving each sample a class based on the kind of extraction method used. Classes attributed were −1, 0 and +1 and specifically +1 to samples obtained by use of ethanol-water mixtures with a higher percentage of ethanol (**A** and **B**), 0 to samples obtained by use of ethanol-water mixtures with a lower percentage of ethanol (**C**–**F**) and −1 to samples obtained by glycerol extraction and without ethanol (**G** and **H**). UV was applied before multivariate data analysis. Models were validated by cross-validation techniques and permutation tests according to standardized good practice to minimize false discoveries and to obtain robust statistical models.

## 4. Conclusions

*Helichrysum italicum* derived food supplements are used for their healthy effects on microcirculation, on the inflammation of the nose and throat, and on digestion and the liver [9,10]. They are distributed both in drug stores and on on-line markets as hydroalcoholic extracts with different percentages of water and as mother tinctures. Each extraction method has its own advantages and disadvantages, but the main goal of the chosen method is the achievement of the complete extraction of the compounds of interest and the avoidance of their chemical modification. In this work, metabolite profiles of derived food supplements of *H. italicum* were obtained by ^1^H NMR experiments and data were processed by multivariate statistical analysis to highlight differences in the commercial products and to evidence those with the highest concentrations of bioactive metabolites. The ^1^H NMR profile of hydroalcoholic extracts and mother tinctures based on *H. italicum* revealed clear differences. It is evident that no qualitative but quantitative variation occurs in its commercial products. In detail, this work highlights how derived food supplements of *H. italicum* obtained using ethanol-water mixtures ranging from 45% to 20% of ethanol represent the products with the highest amount of both primary (amino acids) and secondary metabolites as 3,4-dicaffeoylquinic acid (**9**), chlorogenic acid (**10**), 3,5-dicaffeoylquinic acid (**11**), and kaempferol 3-*O*-glucopyranoside (**12**). Moreover, it is evident that the use of an ethanol-water mixture 20:80, used for the commercial product **F**, is the most suitable method to afford the highest number of phenolic compounds. Food derived supplements obtained by glycerol extraction are characterized by their high amount of β-glucose and α-glucose and a lower content of phenolic compounds. Therefore, the ^1^H NMR analysis followed by multivariate data analysis confirmed its usefulness in herbal product characterization: the importance of detecting compounds accounting for the biological activity with a single experiment is crucial for the differentiation of derived food supplements obtained by green extraction procedures.

## Figures and Tables

**Table 1:** Characteristic ^1^H NMR peaks identified in *H. italicum* food supplements.

	Compound	1H Chemical Shifts (Multiplicity, *J* in Hz)
**1**	alanine	1.48 * (d, 7.2), 3.83 (q, 6.0)
**2**	GABA	1.92 * (t, 7.5), 2.30 (t, 7.3) 3.0 (t, 7.5)
**3**	lysine	1.52 (m), 1.73 (m), 2.00 * (m)
**4**	valine	0.98 (d, 7), 1.03 (d, 7.0) 2.31 * (m)
**5**	12-hydroxytremetone	2.56 * (s), 3.21 (dd, 10.0, 16.0), 3.53 (dd, 10.0, 16.0), 4.20 s, 5.26 (t, 10.0), 5.45 (s), 6.87 (d, 8.0), 7.01 (d, 8.0, 1.2), 7.89 (d, 1.2)
**6**	gnaphaliol	2.60 * (s), 4.25 (d, 13.2), 4.27 (d, 13.2), 5.23 (d, 6.2), 5.29 (d, 6.2), 5.41 s, 6.99 (d, 8.0), 8.04 (d, 8.0, 1.2), 8.12 (d, 1.2)
**7**	β-glucose	4.50 * (d, 8.0)
**8**	α-glucose	5.14 * (d, 3.6)
**9**	3,4-dicaffeoylquinic acid	2.02 (m), 2.09 (m), 2.17 (m), 2.28 (m), 4.32 (m), 5.12 (brd, 6.5); 5.63 (m), 6.29 (d, 16.0), 6.23 * (d, 16.0), 6.76 (d, 8.0), 6.75 (d, 8.3), 6.91 (dd, 8.0, 2.0), 6.92 (dd, 8.3, 2.0), 7.02 (d, 2.0), 7.52 (d, 16.0), 7.60 (d, 16.0)
**10**	chlorogenic acid	2.02 (t, 12.0), 2.15 (t, 12.0); 3.73 (dd, 2.0, 8.5), 4.16 (m), 5.42 (t, 8.5), 6.31 * (d, 16.0), 6.80 (d, 8.0), 6.98 (d, 8.0, 1.2), 7.08 (d, 1.2), 7.60 (d, 16.0)
**11**	3,5-dicaffeoylquinic acid	2.16 (m), 2.21 (m), 2.24 (m), 2.32 (dd, 13.5, 3.5), 3.97 (dd, 7.5, 3.0), 5.38 (m), 5.43 (m), 6.26 (d, 16.0), 6.37 * (d, 16.0), 6.77 (d, 8.0,), 6.78 (d, 8.3), 6.96 (dd, 8.0, 2.0), 6.97 (dd, 8.3, 2.0), 7.06 (d, 2.0, x2), 7.58 (d, 16.0), 7.62 (d, 16.0)
**12**	kaempferol 3-*O*-glucopyranoside	3.21–3.5 (m), 3.69 (dd, 12.0, 2.0), 3.53 (dd, 12.0, 2.0), 6.16 (d, 1.2), 6.35 (d, 1.2), 6.86 (d, 8.2), 7.87 * (d, 8.2)

* Signals chosen for multivariate data analysis.

## Data Availability

The data presented in this study are available in supplementary materials.

## Supplementary Materials

The following materials are available online: Figures S1–S18. ^1^H NMR Spectra (600 MHz, CD3OD) of derived food supplements A–H and HSQC, HMBC and COSY spectra of derived food supplements C, D and F. Figure S19. PCA of *H. italicum* derived food supplements obtained by targeted analysis: PCA single variables to the principal component 1 and 2.

## Sample Availability

Samples of the compounds **1**–**12** are available from the authors.

## References

[1] Benincasa C., Santoro I., Nardi M., Cassano A., Sindona G. (2019). Eco-Friendly Extraction and Characterisation of Nutraceuticals from Olive Leaves. Molecules.

[2] Sampaio B.L., Edrada-Ebel R., Da Costa F.B. (2016). Effect of the environment on the secondary metabolic profile of *Tithonia diversifolia*: A model for environmental metabolomics of plants. Sci. Rep..

[3] Ranjha M.M.A.N., Kanwal R., Shafique B., Arshad R.N., Irfan S., Kieliszek M., Kowalczewski P.Ł., Irfan M., Khalid M.Z., Roobab U. (2021). A Critical Review on Pulsed Electric Field: A Novel Technology for the Extraction of Phytoconstituents. Molecules.

[4] Celeiro M., Garcia-Jares C., Llompart M., Lores M. (2021). Recent Advances in Sample Preparation for Cosmetics and Personal Care Products Analysis. Molecules.

[5] Chemat F., Vian M.A., Cravotto G. (2012). Green extraction of natural products: Concept and principles. Int. J. Mol. Sci..

[6] Bottone A., Masullo M., Montoro P., Pizza C., Piacente S. (2019). HR-LC-ESI-Orbitrap-MS based metabolite profiling of *Prunus dulcis* Mill. (Italian cultivars Toritto and Avola) husks and evaluation of antioxidant activity. Phytochem. Anal..

[7] Cerulli A., Napolitano A., Hosek J., Masullo M., Pizza C., Piacente S. (2021). Antioxidant and In Vitro Preliminary Anti-Inflammatory Activity of *Castanea sativa* (Italian Cultivar “Marrone di Roccadaspide” PGI) Burs, Leaves, and Chestnuts Extracts and Their Metabolite Profiles by LC-ESI/LTQOrbitrap/MS/MS. Antioxidants.

[8] Nutrizio M., Gajdoš Kljusurić J., Marijanović Z., Dubrović I., Viskić M., Mikolaj E., Chemat F., Režek Jambrak A. (2020). The Potential of High Voltage Discharges for Green Solvent Extraction of Bioactive Compounds and Aromas from Rosemary (*Rosmarinus officinalis* L.)—Computational Simulation and Experimental Methods. Molecules.

[9] Mari A., Napolitano A., Masullo M., Pizza C., Piacente S. (2014). Identification and quantitative determination of the polar constituents in *Helichrysum italicum* flowers and derived food supplements. J. Pharm. Biomed. Anal..

[10] Rigano D., Formisano C., Pagano E., Senatore F., Piacente S., Masullo M., Capasso R., Izzo A.A., Borrelli F. (2014). A new acetophenone derivative from flowers of *Helichrysum italicum* (Roth) Don ssp. italicum. Fitoterapia.

[11] Antunes Viegas D., Palmeira-de-Oliveira A., Salgueiro L., Martinez-de-Oliveira J., Palmeira-de-Oliveira R. (2014). *Helichrysum italicum*: From traditional use to scientific data. J. Ethnopharmacol..

[12] Mancini E., De Martino L., Marandino A., Scognamiglio M.R., De Feo V. (2011). Chemical Composition and Possible in Vitro Phytotoxic Activity of *Helichrsyum italicum* (Roth) Don ssp. italicum. Molecules.

[13] Mastelić J., Politeo O., Jerković I. (2008). Contribution to the Analysis of the Essential Oil of *Helichrysum italicum* (Roth) G. Don.—Determination of Ester Bonded Acids and Phenols. Molecules.

[14] Cerulli A., Masullo M., Montoro P., Hosek J., Pizza C., Piacente S. (2018). Metabolite profiling of “green” extracts of *Corylus avellana* leaves by ^1^H NMR spectroscopy and multivariate statistical analysis. J. Pharm. Biomed. Anal..

[15] Bottone A., Montoro P., Masullo M., Pizza C., Piacente S. (2020). Metabolite profiling and antioxidant activity of the polar fraction of Italian almonds (Toritto and Avola): Analysis of seeds, skins, and blanching water. J. Pharm. Biomed. Anal..

[16] Beteinakis S., Papachristodoulou A., Gogou G., Katsikis S., Mikros E., Halabalaki M. (2020). NMR-Based Metabolic Profiling of Edible Olives—Determination of Quality Parameters. Molecules.

[17] Sellami H.K., Napolitano A., Masullo M., Smiti S., Piacente S., Pizza C. (2013). Influence of growing conditions on metabolite profile of *Ammi visnaga* umbels with special reference to bioactive furanochromones and pyranocoumarins. Phytochemistry.

[18] Bottone A., Montoro P., Masullo M., Pizza C., Piacente S. (2018). Metabolomics and antioxidant activity of the leaves of *Prunus dulcis* Mill. (Italian cvs. Toritto and Avola). J. Pharm. Biomed. Anal..

[19] Do Amaral F.P., Napolitano A., Masullo M., dos Santos L.C., Festa M., Vilegas W., Pizza C., Piacente S. (2012). HPLC-ESIMS^n^ Profiling, Isolation, Structural Elucidation, and Evaluation of the Antioxidant Potential of Phenolics from *Paepalanthus geniculatus*. J. Nat. Prod..

[20] Seigner J., Junker-Samek M., Plaza A., D’Urso G., Masullo M., Piacente S., Holper-Schichl Y.M., de Martin R. (2019). A *Symphytum officinale* Root Extract Exerts Anti-inflammatory Properties by Affecting Two Distinct Steps of NF-kappa B Signaling. Front. Pharmacol..

[21] Materska M., Olszowka K., Chilczuk B., Stochmal A., Pecio L., Pacholczyk-Sienicka B., Piacente S., Pizza C., Masullo M. (2019). Polyphenolic profiles in lettuce (*Lactuca sativa* L.) after CaCl_2_ treatment and cold storage. Eur. Food Res. Technol..

[22] Masullo M., Lauro G., Cerulli A., Kontek B., Olas B., Bifulco G., Piacente S., Pizza C. (2021). Giffonins, Antioxidant Diarylheptanoids from *Corylus avellana*, and Their Ability to Prevent Oxidative Changes in Human Plasma Proteins. J. Nat. Prod..

[23] Cerulli A., Lauro G., Masullo M., Cantone V., Olas B., Kontek B., Nazzaro F., Bifulco G., Piacente S. (2017). Cyclic Diarylheptanoids from *Corylus avellana* Green Leafy Covers: Determination of Their Absolute Configurations and Evaluation of Their Antioxidant and Antimicrobial Activities. J. Nat. Prod..

[24] Masullo M., Cerulli A., Mari A., Santos C.C.D., Pizz C., Piacente S. (2017). LC-MS profiling highlights hazelnut (Nocciola di Giffoni PGI) shells as a byproduct rich in antioxidant phenolics. Food Res. Int..

[25] Liu W., Li J., Zhang X., Zu Y., Yang Y., Liu W., Xu Z., Gao H., Sun X., Jiang X. (2020). Current Advances in Naturally Occurring Caffeoylquinic Acids: Structure, Bioactivity, and Synthesis. J. Agric. Food Chem..

[26] Mahmood N., Piacente S., Burke A., Khan A., Pizza C. (1997). Constituents of *Cuscuta reflexa* are anti-HIV agents. Antivir. Chem. Chemother..

[27] Santana-Gálvez J., Cisneros-Zevallos L., Jacobo-Velázquez D.A. (2017). Chlorogenic Acid: Recent Advances on Its Dual Role as a Food Additive and a Nutraceutical against Metabolic Syndrome. Molecules.

