# Supplementary material for: Metabolite Profiling of Helichrysum italicum Derived Food Supplements by 1H-NMR-Based Metabolomics
Source: Molecules. 2021 Oct 31;26(21):6619. doi: 10.3390/molecules26216619 (PMC8588389; doi:10.3390/molecules26216619)
Supplement: Supplementary file 1 [file molecules-26-06619-s001.zip › molecules-1425239-supplementary.pdf]

Supporting information

Metabolite Profiling of *Helichrysum italicum* Derived Food Supplements by ^{1}H-NMR-Based Metabolomics

Antonietta Cerulli, Milena Masullo, and Sonia Piacente*

Dipartimento di Farmacia, Università degli Studi di Salerno, Via Giovanni Paolo II n. 132, 84084 Fisciano, SA, Italy; piacente@unisa.it
* Correspondence: piacente@unisa.it; Tel.: +39-089-969763

Figure S1. ^{1}H NMR Spectrum (600 MHz, CD_3OD) of **A**

Figure S2. ^{1}H NMR Spectrum (600 MHz, CD_3OD) of **B**

Figure S3. ^{1}H NMR Spectrum (600 MHz, CD_3OD) of **C**

Figure S4. HSQC Spectrum (CD_3OD) of **C**

Figure S5. HMBC Spectrum (CD_3OD) of **C**

Figure S6. COSY Spectrum (CD_3OD) of **C**

Figure S7. ^{1}H NMR Spectrum (600 MHz, CD_3OD) of **D**

Figure S8. HSQC Spectrum (CD_3OD) of **D**

Figure S9. HMBC Spectrum (CD_3OD) of **D**

Figure S10. COSY Spectrum (CD_3OD) of **D**

Figure S11. ^{1}H NMR Spectrum (600 MHz, CD_3OD) of **E**

Figure S12. ^{1}H NMR Spectrum (600 MHz, CD_3OD) of **F**

Figure S13. HSQC Spectrum (CD_3OD) of **F**

Figure S14. HSQC Spectrum (CD_3OD) of **F** region 4.5-8.5 ppm

Figure S15. HMBC Spectrum (CD_3OD) of **F**

Figure S16. COSY Spectrum (CD_3OD) of **F**

Figure S17. ^1H NMR Spectrum (600 MHz, CD_3OD) of **G**

Figure S18. ^1H NMR Spectrum (600 MHz, CD_3OD) of **H**

Figure S19. Principal Component Analysis of *H. italicum* derived food supplements obtained by targeted analysis. A) PCA single variables to the principal component 1 (PC1), B) PCA single variables to the principal component 2 (PC2)

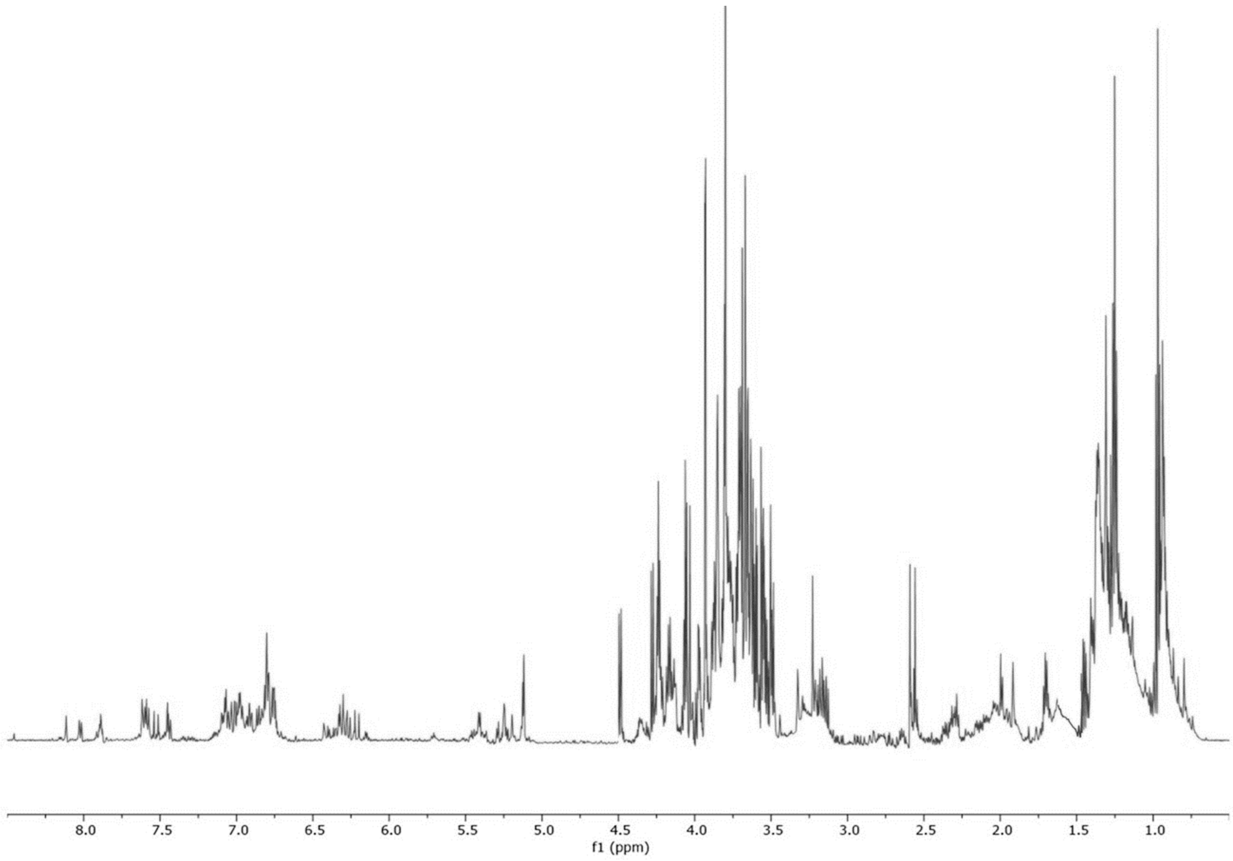

Figure S1. ^{1}H NMR Spectrum (600 MHz, CD_3OD) of **A**

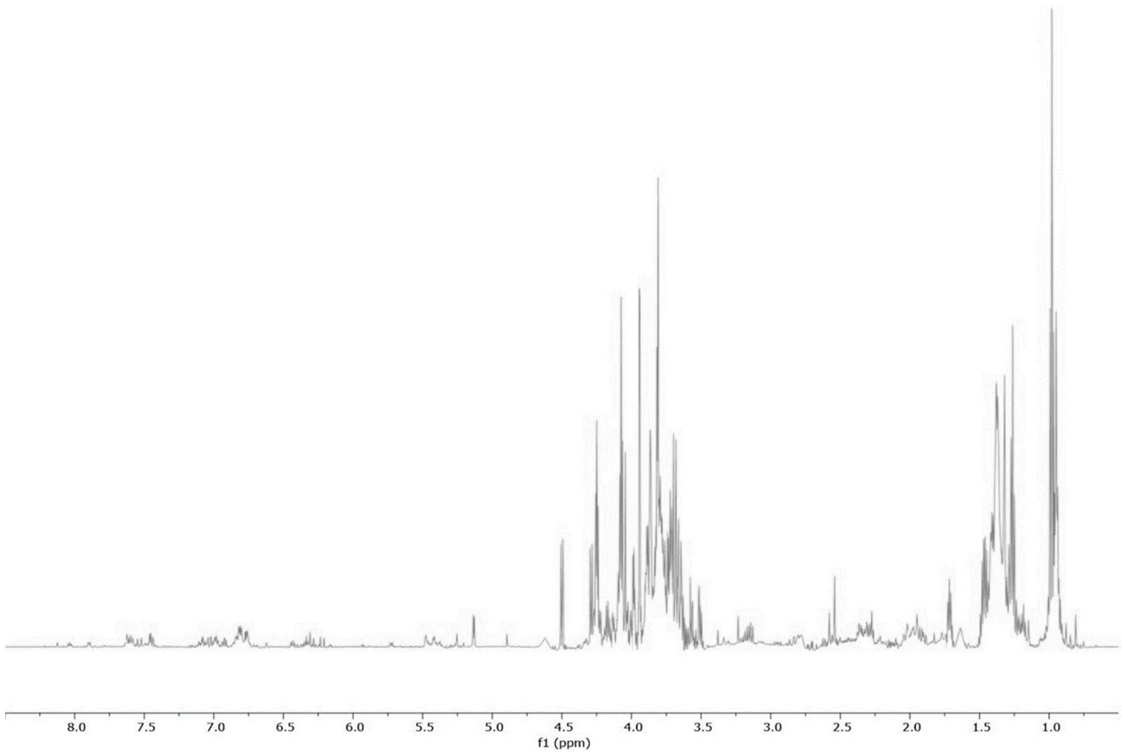

Figure S2. ^{1}H NMR Spectrum (600 MHz, CD_3OD) of **B**

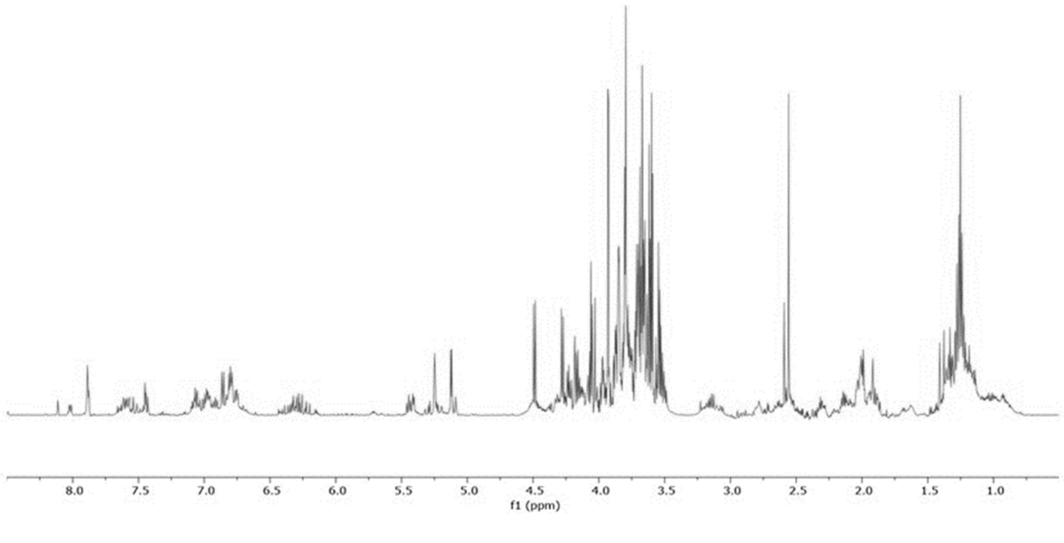

Figure S3. ^{1}H NMR Spectrum (600 MHz, CD_3OD) of **C**

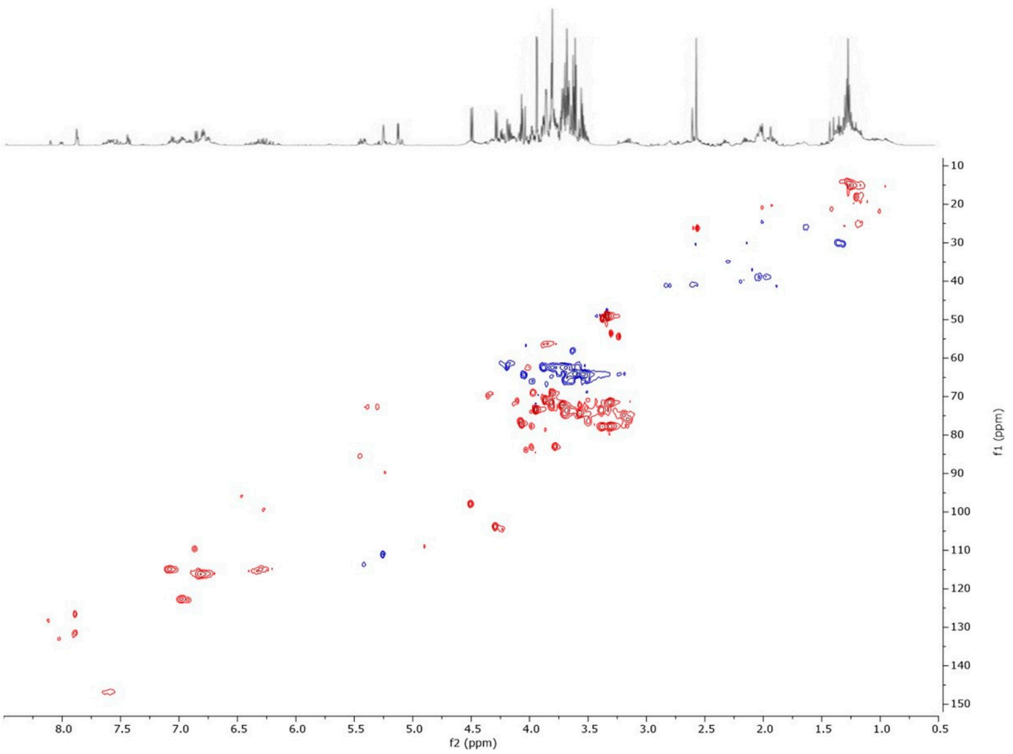

Figure S4. HSQC Spectrum (CD_3OD) of **C**

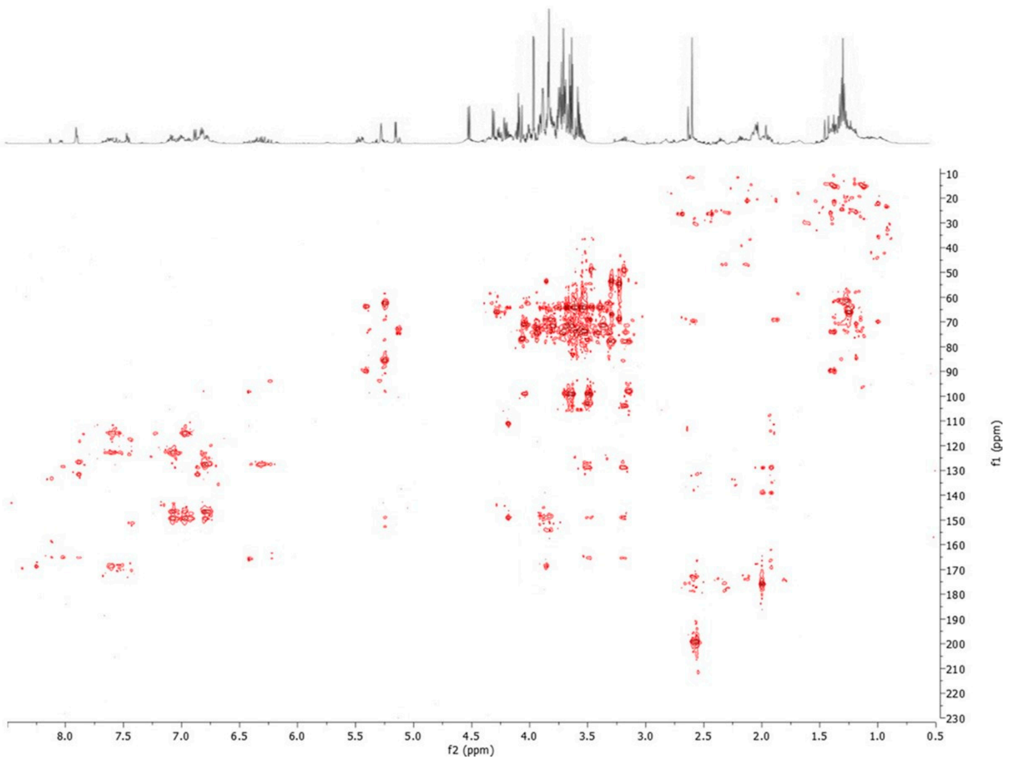

Figure S5. HMBC Spectrum (CD_3OD) of **C**

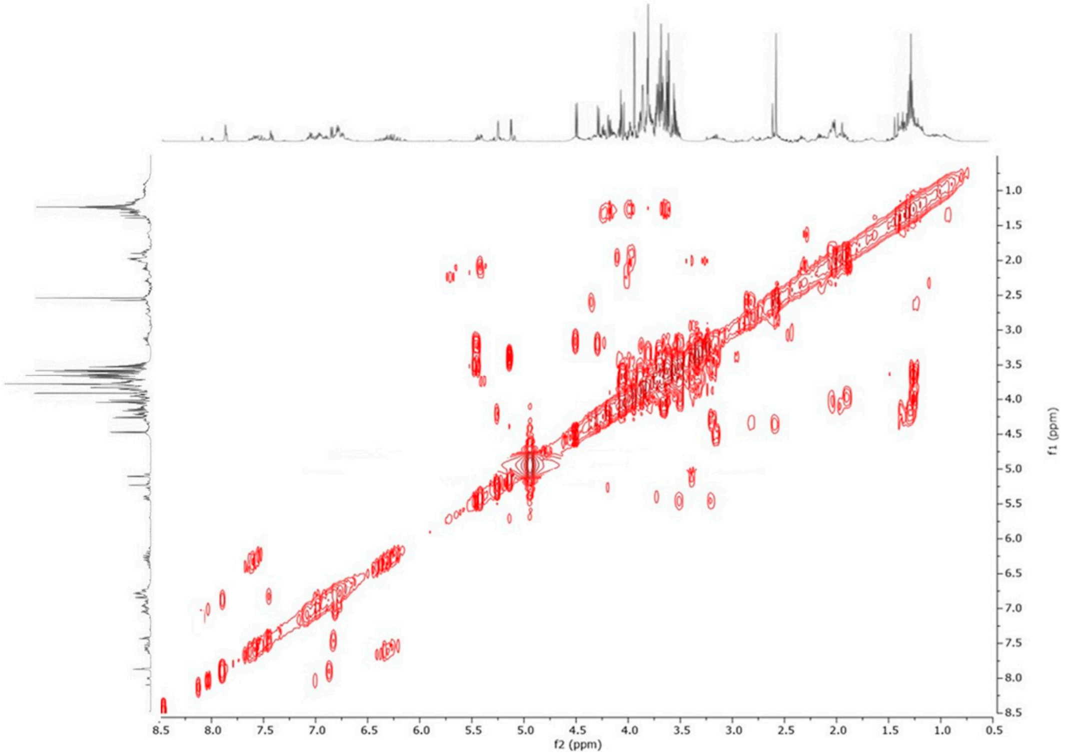

Figure S6. COSY Spectrum (CD_3OD) of **C**

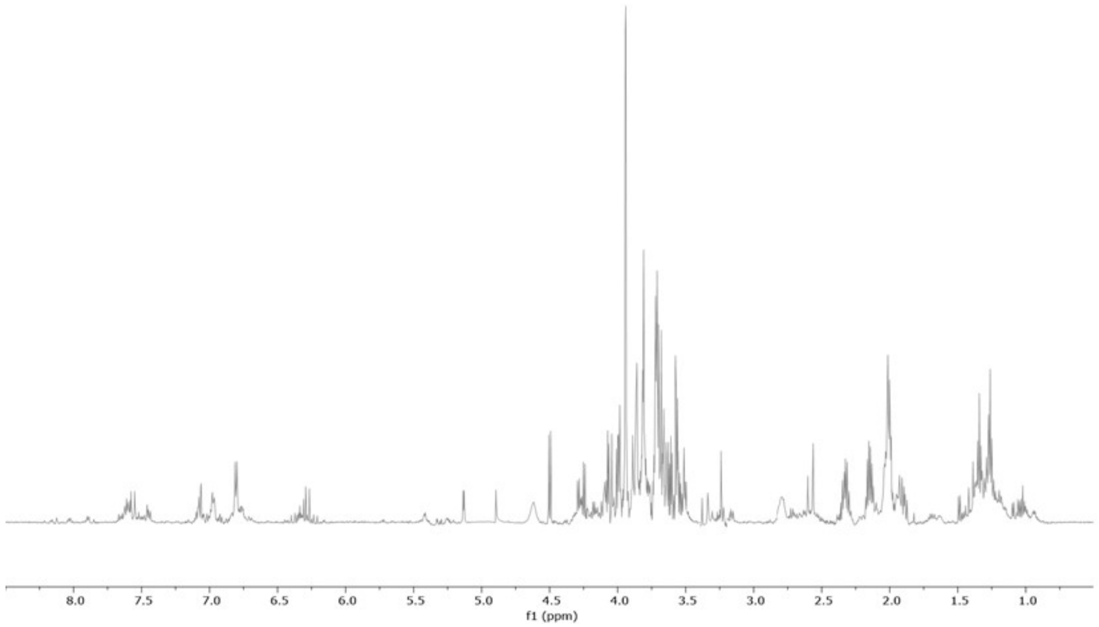

Figure S7. ^1H NMR Spectrum (600 MHz, CD_3OD) of **D**

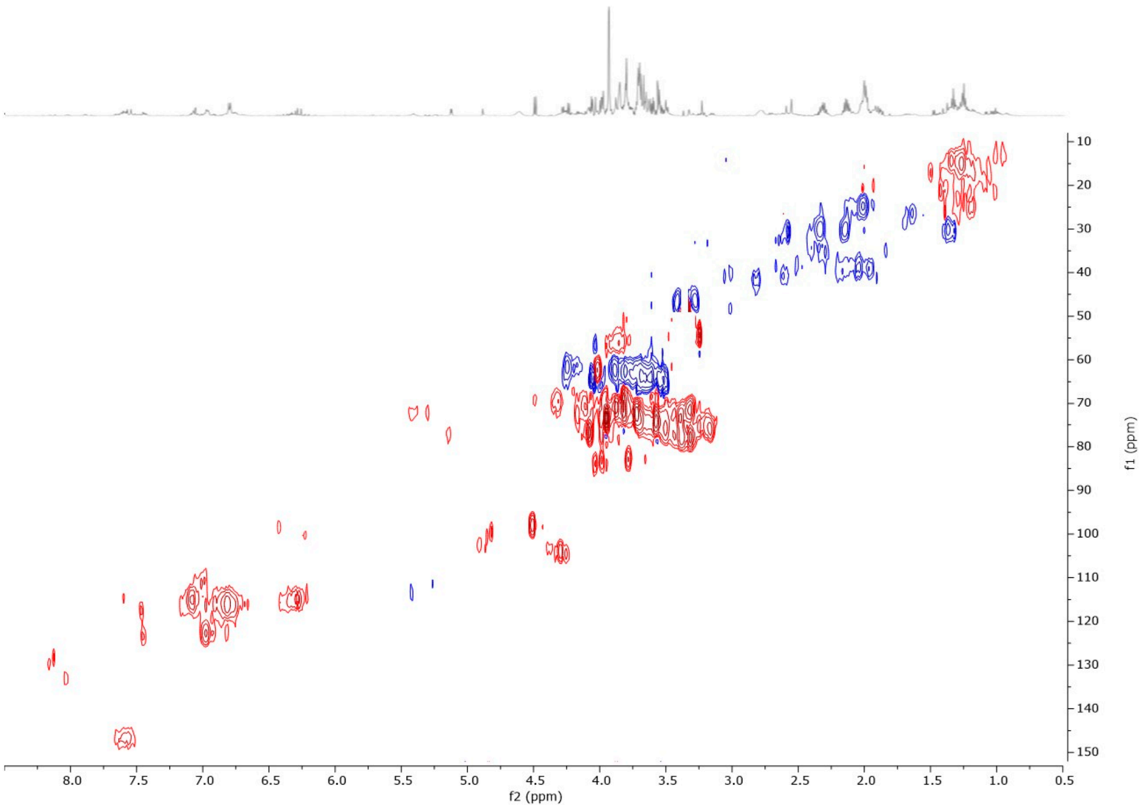

Figure S8. HSQC Spectrum (CD_3OD) of **D**

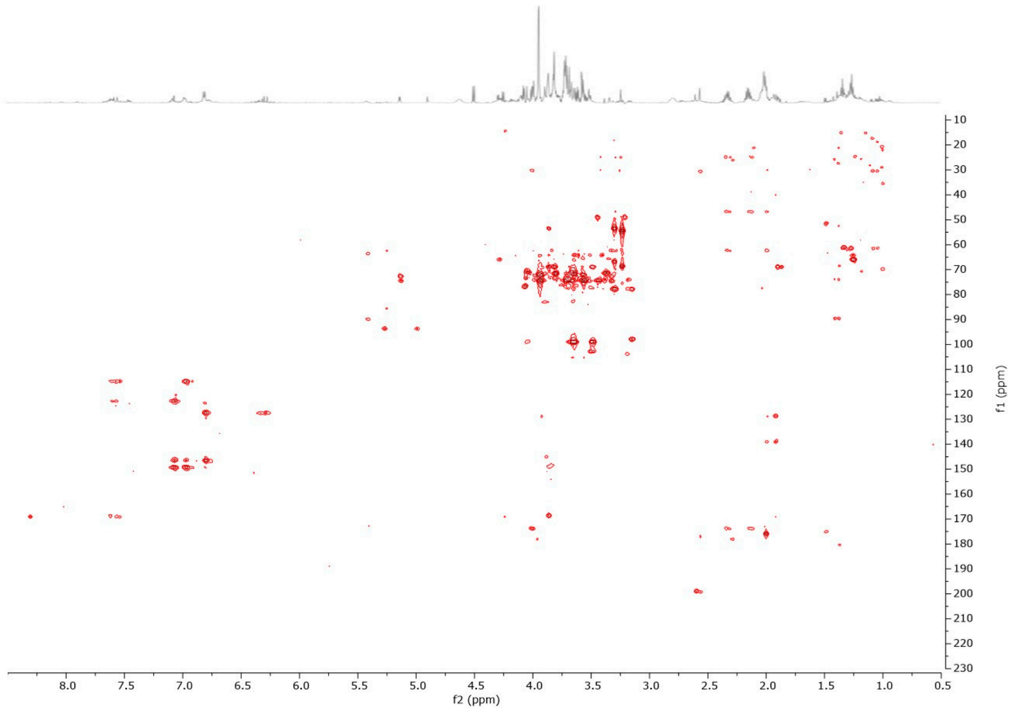

Figure S9. HMBC Spectrum (CD_3OD) of **D**

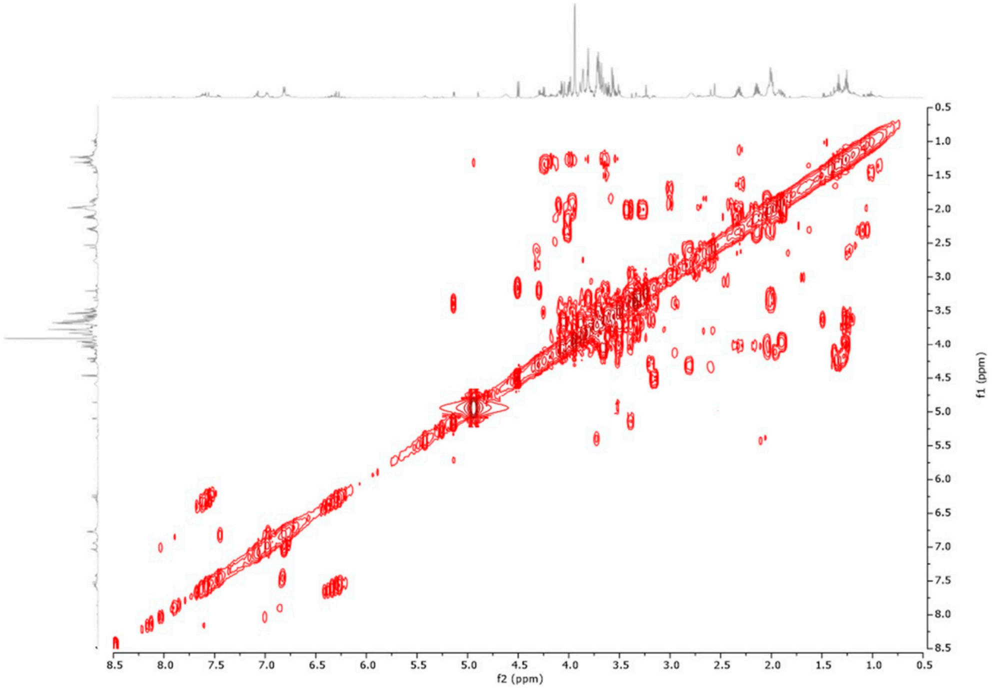

Figure S10. COSY Spectrum (CD_3OD) of **D**

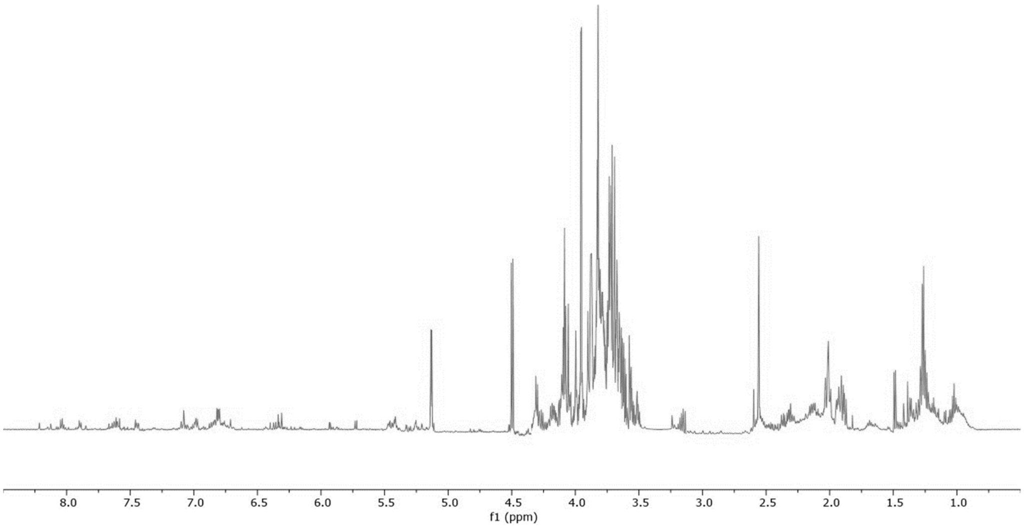

Figure S11. ^1H NMR Spectrum (600 MHz, CD_3OD) of **E**

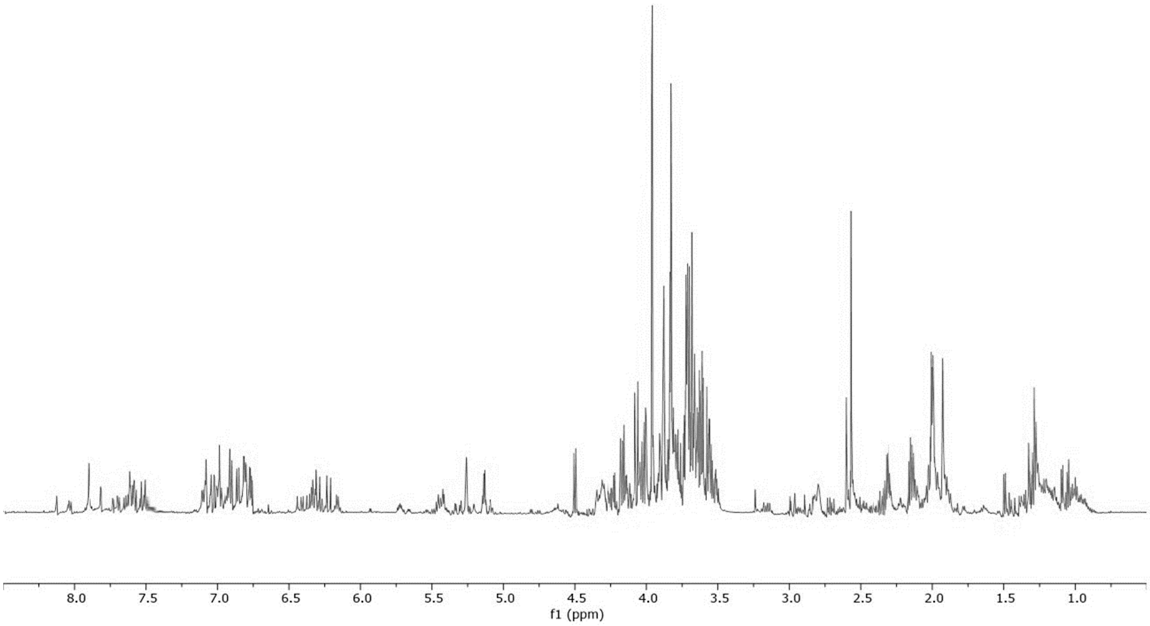

Figure S12. ^{1}H NMR Spectrum (600 MHz, CD_3OD) of **F**

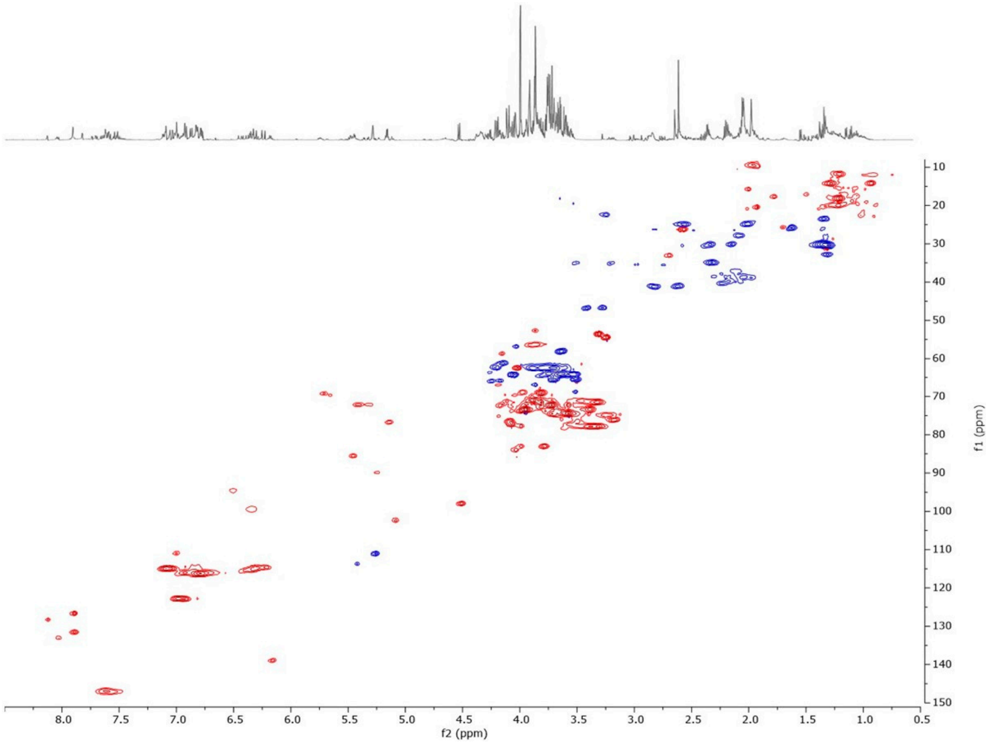

Figure S13. HSQC Spectrum (CD_3OD) of **F**

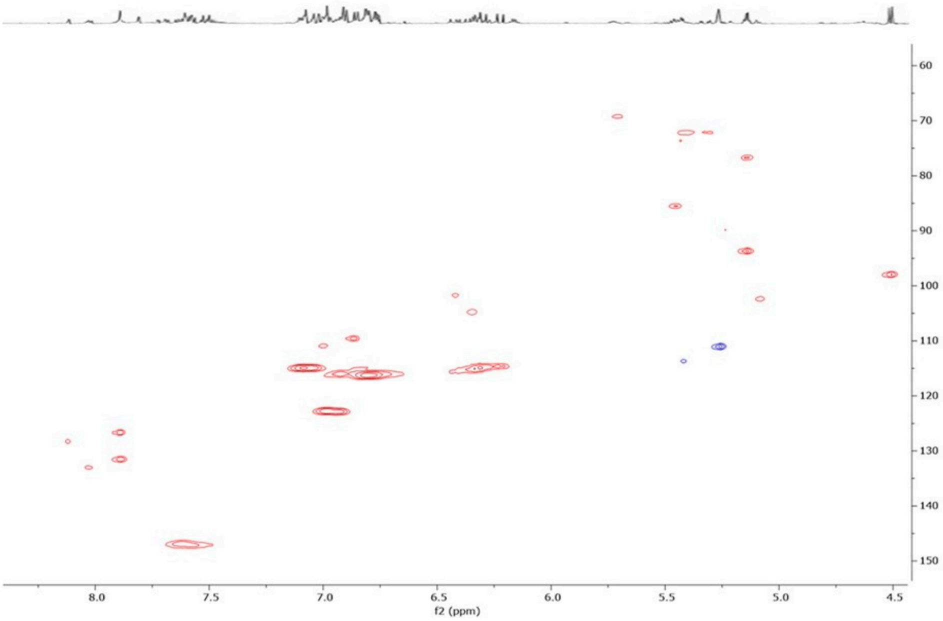

Figure S14. HSQC Spectrum (CD_3OD) of **F** region 4.5-8.5 ppm

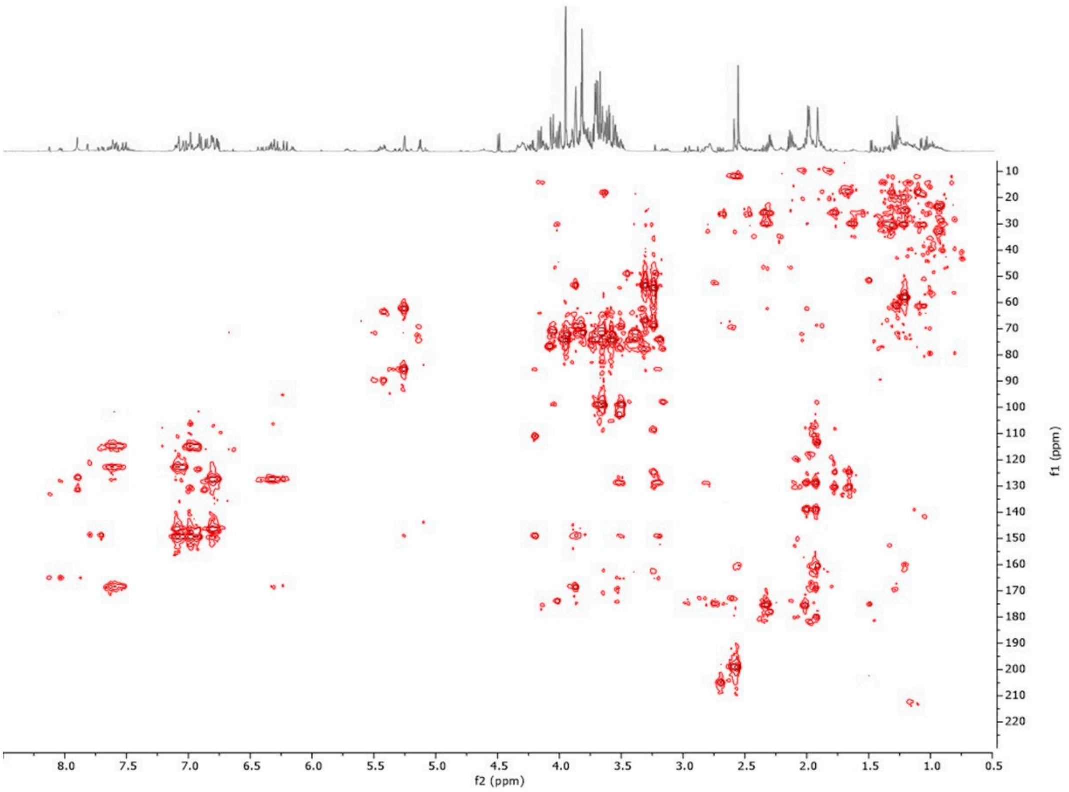

Figure S15. HMBC Spectrum (CD_3OD) of **F**

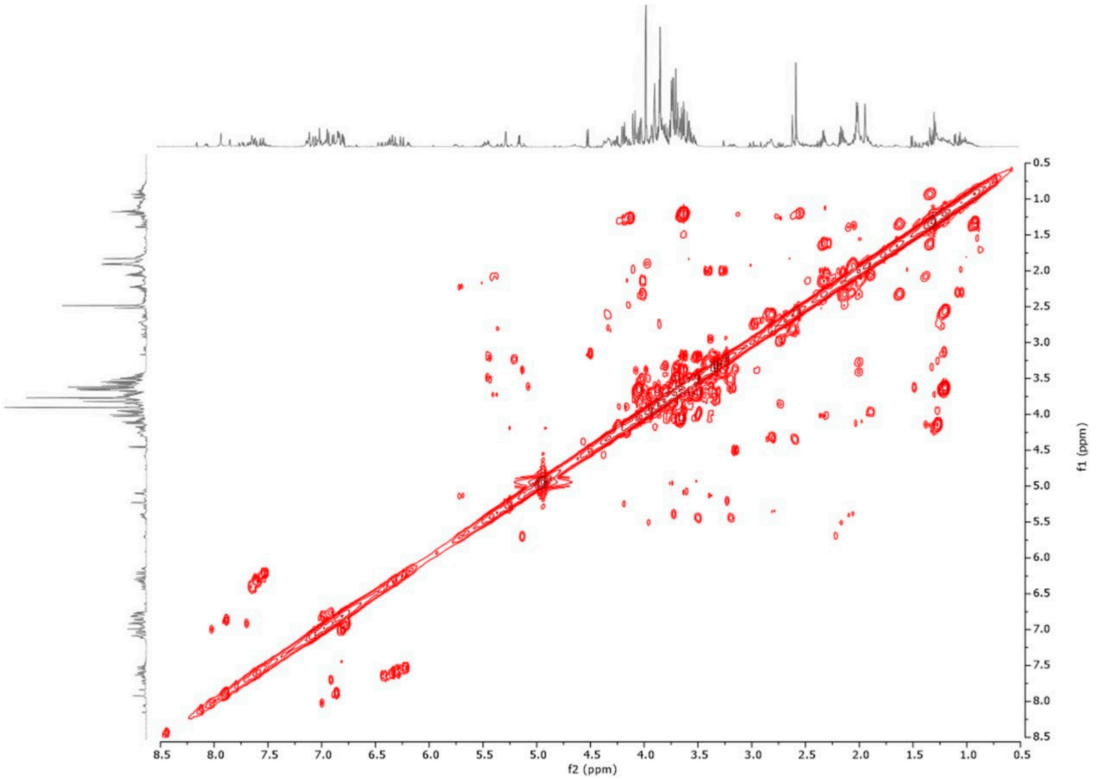

Figure S16. COSY Spectrum (CD_3OD) of **F**

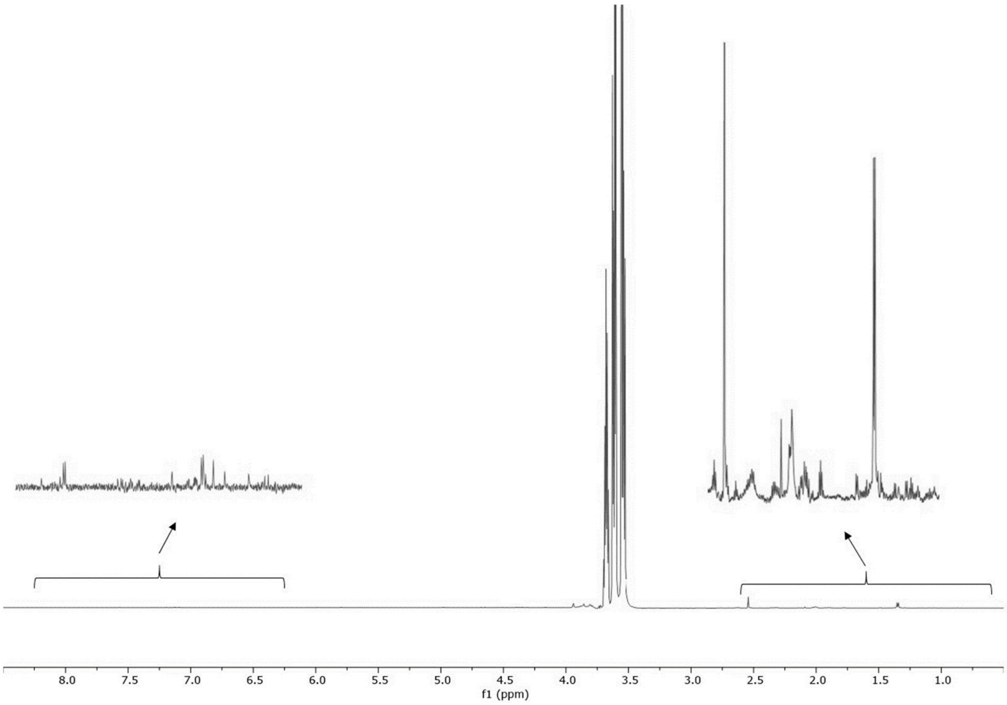

Figure S17. ^{1}H NMR Spectrum (600 MHz, CD_3OD) of **G**

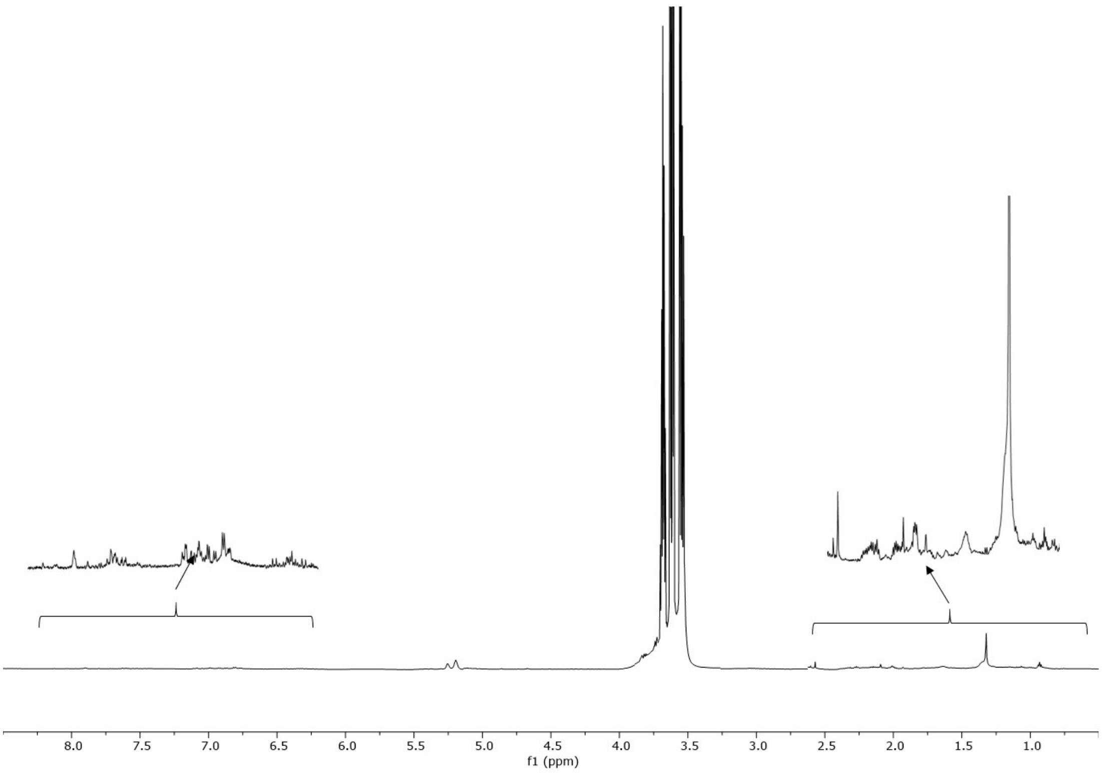

Figure S18. ^{1}H NMR Spectrum (600 MHz, CD_3OD) of **H**

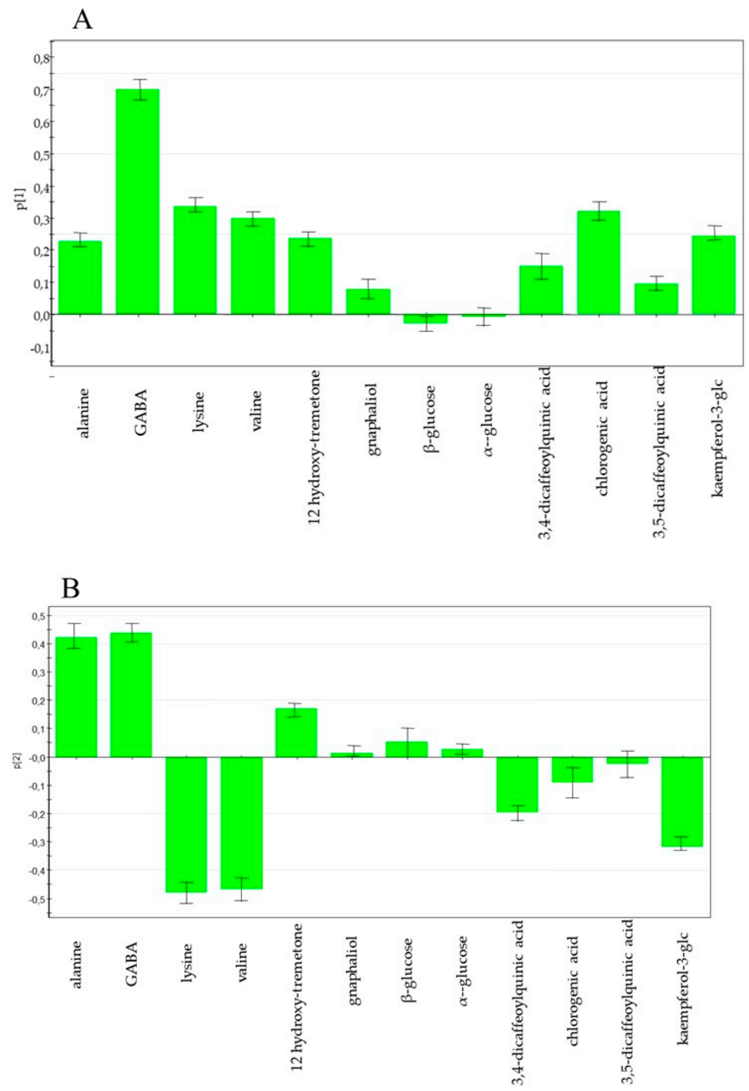

Figure S19. Principal Component Analysis of *H. italicum* derived food supplements obtained by targeted analysis. A) PCA single variables to the principal component 1 (PC1), B) PCA single variables to the principal component 2 (PC2)
